# Association of ankylosing spondylitis with cardiovascular disease: a bidirectional two-sample mendelian randomization study

**DOI:** 10.3389/fgene.2024.1260247

**Published:** 2024-06-26

**Authors:** Pengyu Liu, Juju Shang, Zhi Qi, Shenglei Qiu, Xiaolei Lai, Lixiao Shi, Zhenmin Zhang, Mingxuan Li, Linjing Yang

**Affiliations:** ^1^ Department of Cardiology, Beijing Traditional Chinese Medicine Hospital, Capital Medical University, Beijing, China; ^2^ Department of Ultrasound, Beijing Traditional Chinese Medicine Hospital, Capital Medical University, Beijing, China

**Keywords:** ankylosing spondylitis, cardiovascular diseases, heart failure, mendelian randomization, causality

## Abstract

**Backgrounds:**

Current observational investigations hint at a potential linkage between ankylosing spondylitis and cardiovascular wellness. However, the nature of this causality remains to be elucidated. Consequently, this study is designed to evaluate the causal interconnection between ankylosing spondylitis and cardiovascular-related conditions utilizing a bidirectional two-sample Mendelian Randomization (MR) methodology.

**Methods:**

In this study, we conducted Mendelian randomization (MR) analyses using genome-wide association study (GWAS) data. The fixed-effects inverse variance weighted (IVW) model was used as the primary analysis method, and MR-Egger regression and the weighted median method were employed as supplementary approaches. Horizontal pleiotropy and heterogeneity were evaluated using various statistical tests, including MR-PRESSO global test, MR-Egger intercept, and Cochran’s Q test.

**Results:**

The MR result demonstrated an increased risk of heart failure in individuals with ankylosing spondylitis (OR: 1.0132, 95% CI = 1.0043-1.0221, *p* = 0.003). The MR analysis results did not demonstrate a causal relationship between ankylosing spondylitis and other cardiovascular diseases, such as atrial fibrillation, coronary artery disease, ischemic stroke, myocardial infarction, and valvular heart disease (all *p* > 0.05). No evidence of reverse causality was found between ankylosing spondylitis and mentioned cardiovascular diseases in reverse MR analyses. Sensitivity analysis verified the reliability of the results.

**Conclusion:**

Our MR study indicates a relationship between ankylosing spondylitis and an increased risk of heart failure. Further research is needed to confirm these findings and elucidate the underlying mechanisms involved.

## Introduction

Cardiovascular diseases (CVDs) encompass a multitude of disorders affecting the heart and vascular system (Tsao et al., 2022). Both genetic and environmental factors may play a role in the development and progression of CVDs. Despite advances in diagnosing and treating these disorders, CVDs remain the principal cause of mortality and disability worldwide (Roth et al., 2020). Given the severe societal and clinical consequences, early identification and intervention for CVDs risk factors are vital for reducing incidence and mortality rates (Roger et al., 2020). Notably, some studies suggest a possible association between ankylosing spondylitis and CVDs (Ding et al., 2022; Kwon et al., 2022).

Ankylosing spondylitis (AS) is classified as an autoimmune disease leading to bone remodeling and spinal rigidity (Toussirot, 2021). Previous studies have not definitively established the risk of CVDs in patients with AS. While some studies indicate AS as an independent risk factor for CVDs (Setyawan et al., 2021; Kwon et al., 2022), conflicting results have been published (Tsai et al., 2015), and whether these relationships are causal and their directionality remain unclear. Recently, a large meta-analysis indicated that CVD comorbidities are common in AS, providing crucial evidence of an association and attracting significant attention (Zhao et al., 2020). Due to the potential limitations of confounding factors and reverse causality in current observational studies, there is an urgent need to explore the causal relationship between AS and CVDs using robust research methods.

Mendelian randomization (MR) is a research method that utilizes genetic variations associated with the exposure of interest as instrumental variables (IVs) to infer the linkages between exposure and disease consequences (Burgess and Thompson, 2015). Mendelian randomization (MR) analyses are less susceptible to confounding, reverse causality, and measurement error compared to conventional observational studies due to the random allocation of genetic variants at conception, which precedes the onset of disease (Lawlor et al., 2008; Yarmolinsky et al., 2019). The applicability of MR studies has been demonstrated in the evaluation of diverse causal relationships between behavioral exposures, educational attainment, socioeconomic statuses, and various diseases (Tillmann et al., 2017; Harrison et al., 2020). Furthermore, prior MR explorations have scrutinized the causal implications of AS on stroke (Mei et al., 2022) and atrial fibrillation (Chen et al., 2022). Nonetheless, these endeavors have largely focused on specific forms of CVDs. Therefore, our research engaged a MR design to ascertain the existence of a causal association between AS and CVDs, thus providing a scientific groundwork for the primary prevention of CVDs.

## Materials and methods

### Study design and GWAS datasets

**FIGURE 1:**
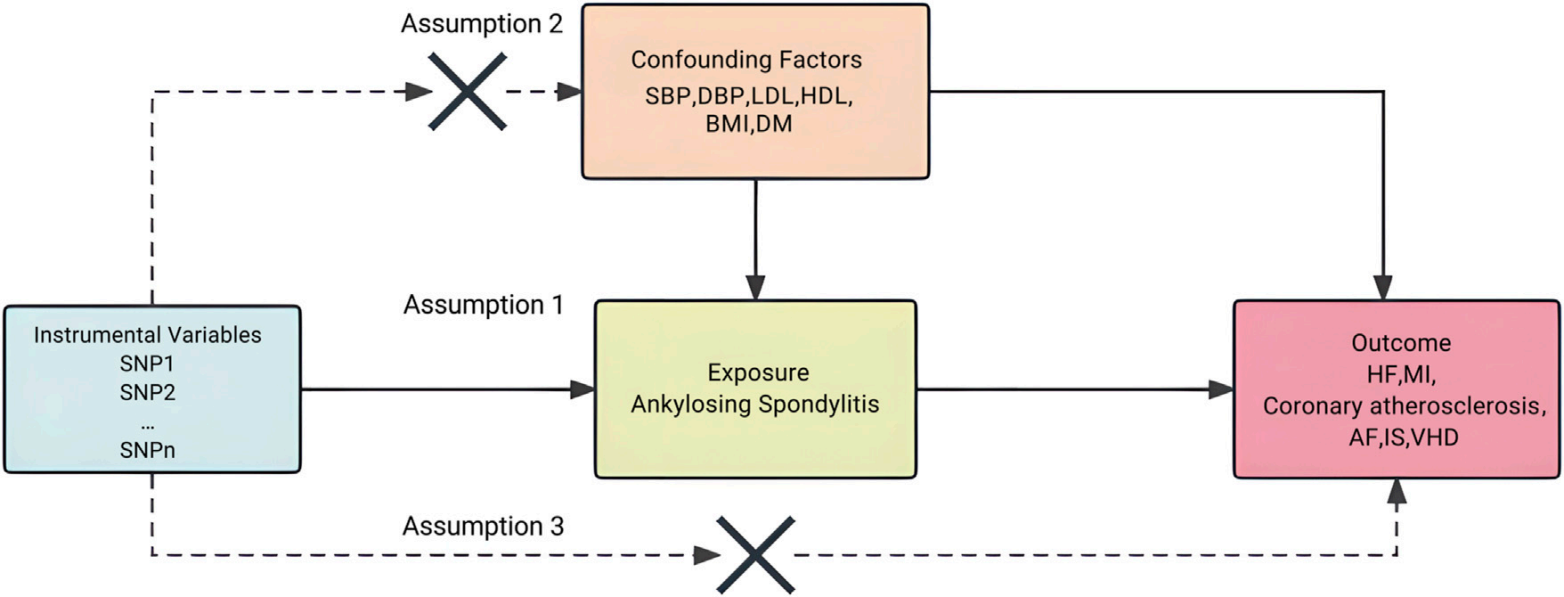
Study design of forward Mendelian randomization. Conceptual schematic of the two-sample Mendelian randomization for the association between ankylosing spondylitis and the risks of cardiovascular disease.

We adopted a two-sample bidirectional Mendelian Randomization (MR) approach to assess the causal relationship between AS and CVDs, the latter specifically encompassing Heart Failure (HF), Coronary Atherosclerosis, Valvular Heart Disease (VHD), Atrial Fibrillation (AF), Myocardial Infarction (MI), and Ischemic Stroke (IS). Concurrently, a reverse analysis was conducted to investigate the potential causal relationship between CVDs and AS. The core premises of our MR design are predicated on three assumptions (Tsao et al., 2022): genetic variations display significant associations with the exposure (Roth et al., 2020); genetic variations are independent of any confounding variables (Roger et al., 2020); genetic variations link to the clinical outcome solely via the exposure path (Davies et al., 2018; Tsao et al., 2022) (Figure 1).

Summary statistic data for AS and VHD were obtained from the FinnGen database (https://www.finngen.fi/en). The datasets (Table 1) for AS and VHD respectively included 166,144 participants (consisting of 1,462 cases and 164,682 controls) and 218,792 participants (including 38,209 cases and 180,583 controls). Data on HF was collected from the HERMES consortium, encompassing 977,323 individuals of European descent (with 47,309 cases and 930,014 controls) (Shah et al., 2020). The summary statistics for AF were derived from a large-scale GWAS meta-analysis, incorporating 60,620 AF cases and 970,216 controls (Nielsen et al., 2018). The summary statistics for Ischemic Stroke were sourced from a large-scale GWAS, including 440,328 individuals of European descent (comprising 34,217 Ischemic Stroke cases and 406,111 controls) (Malik et al., 2018). The GWAS summary statistics for Coronary Atherosclerosis were procured from the UK Biobank, screening 361,194 Europeans (with 14,334 cases and 346,860 controls). The summary statistic data for Myocardial Infarction was obtained from a large-scale GWAS that involved a total of 395,795 participants (includes 14,825 MI cases and 380,970 controls) (Hartiala et al., 2021). To address potential biases resulting from population stratification, the GWAS datasets utilized in this study exclusively included individuals of European ancestry. The current analysis did not require ethical approval as all GWAS statistical data in current analysis are publicly available and have already received approval from relevant ethics review boards.

**TABLE 1:** Sample characteristics for exposures and outcomes in the Mendelian randomization analysis.

Trait	Sample size case/Control	Years	Population	Consortium	Data sources
Ankylosing spondylitis	1,462/164,682	2021	European	FinnGen	finn-b-M13_ANKYLOSPON
Heart Failure	47,309/930,014	2020	European	HERMES	ebi-a-GCST009541
Coronary atherosclerosis	14,334/346,860	2018	European	United Kingdom Biobank	ukb-d-I9_CORATHER
Ischemic stroke	34,217/406,111	2018	European	Malik et al.	ebi-a-GCST006908
Valvular heart disease	38,209/180,583	2021	European	FinnGen	finn-b-I9_VHD_EXNONE
Atrial fibrillation	60,620/970,216	2018	European	Nielsen et al.	ebi-a-GCST006414
Myocardial Infarction	14,825/380,970	2021	European	Hartiala et al.	ebi-a-GCST011364

### Statistical analysis

**FIGURE 2:**
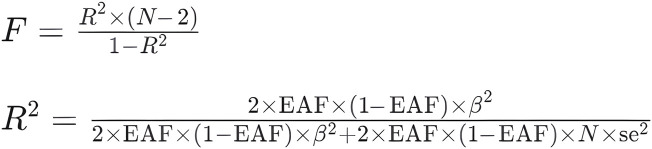
Calculation formula. F: F-statistic, which measures the overall significance of the regression model. R2: Coefficient of determination, which represents the proportion of the variance in the dependent variable explained by the independent variable(s). N: Sample size, the number of observations in the dataset. EAF: Exposure allele frequency, the frequency of the allele associated with the exposure variable in the population. Beta: Regression coefficient, represents the change in the dependent variable associated with a one-unit change in the independent variable. se: Standard error, which measures the variability or uncertainty associated with the estimated regression coefficient.

**FIGURE 3:**
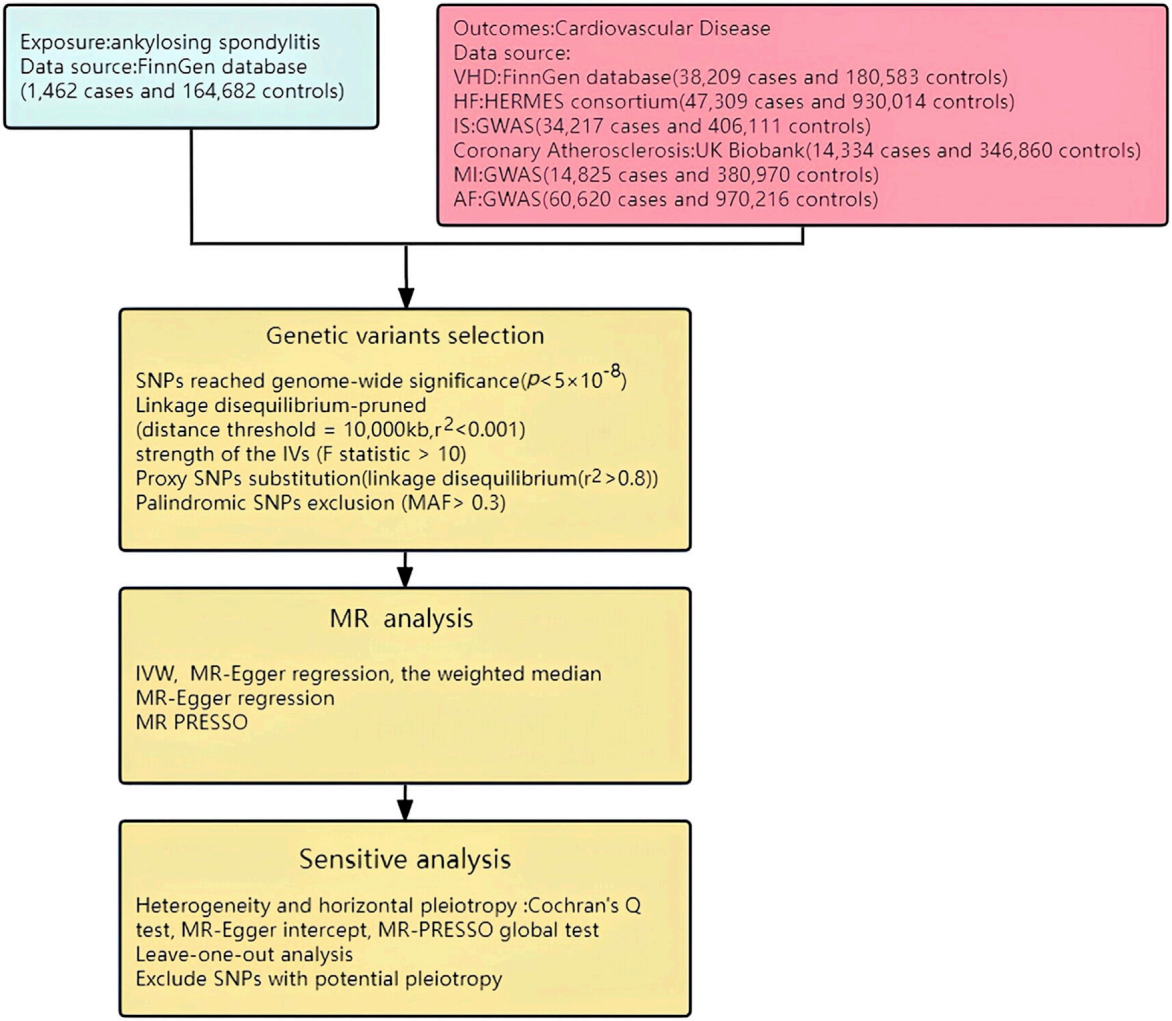
Study flow chart. HF: Heart Failure; MI: Myocardial Infarction; AF: Atrial fibrillation; IS: Ischemic stroke; VHD: Valvular heart disease.

This study was orchestrated in accordance with the STROBE-MR guidelines (Skrivankova et al., 2021a). Initially, we designated single nucleotide polymorphisms (SNPs) with substantial associations with AS as IVs (*p* < 5.0 × 10^−8^). Subsequently, to ensure the independence of genetic variants, a further pruning step was conducted on SNPs based on linkage disequilibrium (LD) (distance threshold = 10,000 kb, *r* ^2^ < 0.001). The F statistic was used to assess the strength of the Ivs in the analysis. An F statistic >10 (Burgess and Thompson, 2011; Pierce et al., 2011) indicates a low risk of weak instrument bias in the MR analysis (the calculation formula can be found in (Figure 2). If the chosen SNP was not included in the GWAS of the outcome variable, LD proxy SNPs from the 1000 Genomes Project (*r* ^2^ > 0.8) were selected as substitutes. Additionally, any palindromic SNP with a minor allele frequency (MAF> 0.3) was removed to ensure the effects of the SNP on exposure and disease are attributed to the same allele (Davey Smith and Hemani, 2014; Burgess et al., 2016). Moreover, to minimize the influence of confounding factors on the outcome variable, we excluded potential instrumental variables (IVs) that were associated with known confounders referring to the human genotype-phenotype association database (PhenoScanner-V2, http://www.phenoscanner.medschl.cam.ac.uk/) (Staley et al., 2016; Kamat et al., 2019). These rigorous steps were taken to ensure the validity and integrity of the IVs used in MR analysis (Figure 3).

The Inverse Variance Weighted (IVW) method was the main analytical approach used in this study. This method combines the Wald ratio estimates of each SNP on the outcome to obtain a summary causal estimate and provide maximum statistical power (Smith and Ebrahim, 2003; Pagoni et al., 2019). In conjunction, we applied the MR-Egger method (Burgess and Thompson, 2017) and the Weighted Median (WM) method (Bowden et al., 2016) as supplementary analysis tools, acknowledged as some of the most scientifically robust and commonly employed methods in this field (Skrivankova et al., 2021b). The assessment of horizontal pleiotropy was conducted by estimating the intercept term derived from the MR-Egger regression, indicating potential bias when the intercept deviates from 0 (Bowden et al., 2015). This method maintains the capacity to generate valid causal estimates even when all IVs are invalid. Compared to the MR-Egger method, the weighted median estimator can identify causal effects even if up to half of the IVs are invalid.

Consequently, the Cochran’s Q test was utilized to assess heterogeneity, with a significance level of *p* < 0.05 indicating the presence of heterogeneity. (Bowden et al., 2019). Horizontal pleiotropy was examined using the MR-Egger intercept test and MR-PRESSO global test (Rees et al., 2017; Verbanck et al., 2018). Prior to MR analysis, the MR-Pleiotropy Residual Sum and Outlier (MR-PRESSO) method is utilized to discard any outliers suspected of potential pleiotropy, ensuring the reliability of MR estimates (Bowden et al., 2017; Verbanck et al., 2018). Moreover, to evaluate the reliability and stability of the MR results, we carried out a sensitivity analysis using the “leave-one-out” method (Hemani et al., 2018). The significance threshold in MR analysis was set as a two-sided *p*-value <0.008 (0.05/6), according to the Bonferroni correction method. *p* values <0.05 but higher than the Bonferroni correction threshold are deemed as potential associations. A reverse MR study assessed the relationships between six CVDs and AS using identical methodologies. R software version 4.0.5, utilizing the “TwoSampleMR” and “MR-PRESSO” packages, was employed for data processing and visualization.

### IV selection and verification

In this study, we obtained 13 significant and independent SNPs as AS SNPs. No SNPs related to cardiovascular disease confounders were found after retrieval on the PhenoScanner website. We found related proxy SNPs in GWAS data to replace a small number of SNPs. In addition, palindromic SNPs with intermediate allele frequencies and SNPs with incompatible alleles were excluded in the harmonization process to eliminate any potential bias. This left 10, 11, 11, 12, 11, 13 SNPs as IVs for causal inference of AS to HF, MI, Coronary Atherosclerosis, AF, IS, and VHD, respectively. Furthermore, the estimation of the F statistic suggests that we did not use weak IVs in the MR analysis (all F statistics >10). The remaining SNPs were chosen as IVs (Supplementary Table S1).

## Results of the MR study

**FIGURE 4:**
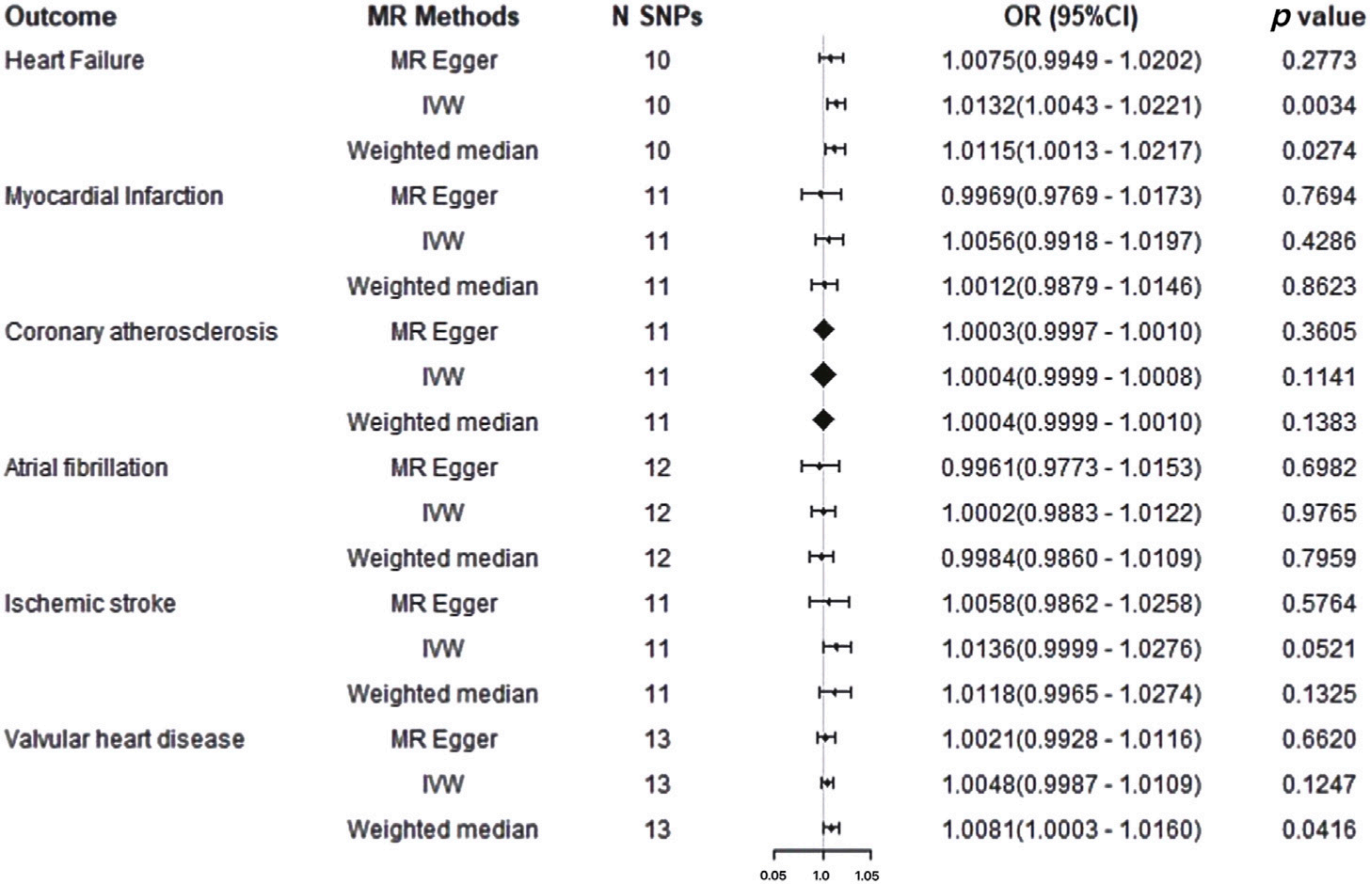
Mendelian randomization results for cardiovascular disease on ankylosing spondylitis risk. IVW: Inverse Variance Weighted method; MR, Mendelian randomization; SNPs, single nucleotide polymorphisms; OR, odds ratio; CI, confidence interval.

In the forward two-sample MR analysis, we utilized IVW, WM, and MR-Egger methods to assess the presence of a causal association between AS and CVDs. Using the fixed effect IVW model as the primary analytical standard an applying Bonferroni correction, we found that Ankylosing Spondylitis amplified the genetic susceptibility risk of HF in the European population (OR = 1.0132, 95% CI 1.0043-1.0221, *p* = 0.0034) (Supplementary Figures SF1–SF3). The WM method (OR = 1.0115, 95%CI = 1.0013–1.0217, *p* = 0.0274) indicated a potential association with a consistent direction. However, the MR-Egger regression model (OR = 1.0075, 95%CI = 0.9949–1.0202, *p* = 0.2773) did not exhibit statistically significant differences (Figure 4). The WM results also suggested a potential association between AS and VHD risk in the European population (OR = 1.0081, 95% CI 1.0003–1.0160, *p* = 0.0416), while IVW and MR-Egger indicated an insignificant association, albeit in a consistent direction. Furthermore, no evidence was found for a potential causal relationship between the genetic susceptibility of Ankylosing Spondylitis and the risk of MI, Coronary atherosclerosis, AF, IS (all *p* > 0.05), with IVW, WM, and MR-Egger results demonstrating consistency (Supplementary Table S2). However, this analysis might lack sufficient statistical power to detect such a weak association.

### Results of sensitivity analysis in MR study

In MR analysis, Cochran’s Q test demonstrated no significant heterogeneity among the IVs for HF, MI, Coronary Atherosclerosis,AF, IS, and VHD, indicating that a fixed effect IVW model is appropriate for analysis. Importantly, no evidence of horizontal pleiotropy was found based on the results of the MR-Egger intercept test and MR-PRESSO test. Furthermore, the leave-one-out analysis demonstrated the robustness of our study, as the exclusion of any individual IV did not significantly impact the overall findings (Supplementary Figure SF4). The MR-PRESSO test did not detect any outliers, which further strengthens the reliability of the results. In summary, the sensitivity analysis affirmed the robustness of our conclusions (Table 2). Furthermore, the funnel plot analysis revealed that, apart from HF and AF, the variation in effect sizes around the point estimates was symmetrical, indicating the absence of substantial evidence for horizontal pleiotropy (Supplementary Figure SF5).

**TABLE 2:** Sensitivity analysis of the MR analysis results of exposures and outcomes.

Outcomes	Pleiotropy test MR-egger			Heterogeneity test MR-egger		IVW^c^		MR-PRESSO global test	
	Intercept	SE^a^	*p*-value^b^	Cochran’s Q	*p*-value	Cochran’s Q	*p*-value	RSS obs^d^	*p*-value
HF^e^	0.0076	0.0062	0.253	1.518	0.992	3.034	0.963	9.752	0.715
MI^f^	0.0109	0.0095	0.282	14.327	0.111	16.409	0.089	21.604	0.144
Coronary atherosclerosis	4.073 × 10^−5^	0.0003	0.902	9.151	0.423	9.168	0.516	9.729	0.726
AF^g^	0.0043	0.0078	0.595	18.986	0.040	19.559	0.052	22.242	0.151
IS^h^	0.0092	0.0087	0.315	11.031	0.274	12.420	0.258	13.919	0.409
VHD^*9^	0.0041	0.0057	0.487	10.709	0.468	11.226	0.510	14.288	0.603

^a^ SE, standard error.

^b^ *p*-value <0.05 was considered as with statistical differences in both heterogeneity and horizontal pleiotropy tests.

^c^ IVW, inverse variance weighting.

^d^ RSS, residual sum of squares.

^e^ HF, Heart Failure.

^f^ MI, Myocardial Infarction.

^g^ AF, Atrial fibrillation.

^h^ IS, Ischemic stroke.

^i^ VHD, valvular heart disease.

### Results of reverse MR study and sensitivity analysis

The results of the fixed effect IVW model did not show potential statistical differences between Heart Failure (OR: 0.8361, 95% CI = 0.4816-1.4517, *p* = 0.5249), Myocardial Infarction (OR: 1.0936, 95% CI = 0.8894-1.3448, *p* = 0.3960), Coronary atherosclerosis (OR: 5.4668, 95% CI = 0.0650-459.6306, *p* = 0.4525), Atrial fibrillation (OR: 0.9994, 95% CI = 0.8962-1.1145, *p* = 0.9917), Ischemic stroke (OR: 0.8816, 95% CI = 0.6030-1.2888, *p* = 0.5153), Valvular heart disease (OR: 0.8449, 95% CI = 0.4052-1.7619, *p* = 0.6532) and AS. The outcomes derived from the MR-Egger regression model and the WM model align with these conclusions (Supplementary Table S3).

In sensitivity analysis, apart from MR-PRESSO, the MR-Egger regression test did not reveal any evidence of pleiotropy effects (Supplementary Table S4). Notably, according to Cochran’s Q test, the causal relationship between MI and AS showed significant heterogeneity; however, random effects IVW produced ineffective results (OR: 1.0936, 95% CI = 0.8894-1.3447, *p* = 0.3960). No significant correlations were found between any other cardiovascular disease and AS. The reverse MR analysis did not yield a significant causal impact of CVD on AS.

## Discussion

In the present investigation, we carried out a bidirectional two-sample MR analysis, utilizing large-scale, publicly available genomic summary data to scrutinize the potential causal impacts of Ankylosing Spondylitis on six distinct CVDs. Our data provide evidence of a significant causal association between AS and the risk of HF, but no causal association between AS and susceptibility to other CVDs. Additionally, the reverse SVMR study suggests no relevance of high risk of HF, MI, Coronary atherosclerosis, AF, IS, and VHD to AS. In summary, the current study results suggest that patients with ankylosing spondylitis should be vigilant about the increased risk of HF and take preventive measures as early as possible. Furthermore, this topic should be further validated in large-scale studies in the future, deepening the disease typing and severity of heart failure, as well as assessing treatment options for patients with cardiac involvement.

Our study findings strengthen or extend the existing observational evidence. A longitudinal cohort study involving 77,928 participants pointed out an increased incidence of congestive HF in patients with AS (Bae et al., 2019), which is largely consistent with our results. Another study showed that the correlation between AS and heart failure varies among populations, with the risk of HF significantly higher in the male AS cohort aged 60 to 69 than in the general population cohort (Hung et al., 2016). It is noteworthy that these cohort studies can only provide associative evidence, unable to determine the causality between the two. In addition, another systematic review of echocardiographic features in patients with AS indicates an increased risk of diastolic left ventricular dysfunction in AS patients, with specific electrocardiographic manifestations of poorer E/A ratio, longer deceleration time, and prolonged average isovolumic relaxation time (Heslinga et al., 2014). Further research found that TNF blockade therapy may improve several echocardiographic parameters of cardiac function in AS patients, and the level of NT-proBNP improves after using TNF blockade therapy in AS (Heslinga et al., 2015). However, these results focus on echocardiographic parameters and biochemical indicators, the clinical significance of which is unclear.

In the past decade, several observational studies exploring the relationship between chronic AS and CVD have yielded conflicting results. For example, while most cohort studies found an increased risk of ischemic heart disease in patients with AS (Szabo et al., 2011; Huang et al., 2013; Wright et al., 2015), a study by Essers et al. did not support this conclusion (adjusted HR 1.20 (95% CI 0.97–1.48)) (Essers et al., 2016). A meta-analysis published by Turina et al., in 2011 showed that the incidence of MI in AS patients was 7.4%, compared to 4.6% in the control group, and the incidence of stroke in AS patients was 2.2%, compared to 2.3% in the control group, with none of the meta-analysis results reaching a significant level (So et al., 2017). In addition, a retrospective cohort study in 2012 involving 1,686 AS patients found a risk ratio of 1.28 for MI and 1.0 for CVD/stroke, again not reaching a significant level (Brophy et al., 2012). Moreover, non-ischemic cardiac manifestations such as arrhythmias (Forsblad-d'Elia et al., 2013; Bengtsson et al., 2019), valvular diseases (Palazzi et al., 2008), and diastolic dysfunction (Heslinga et al., 2014) have also been observed in AS. Finally, AS has been associated with cardiovascular risk factors such as diabetes (Chen et al., 2014; Chou et al., 2014), hypertension (Chen et al., 2014; Chou et al., 2014), dyslipidemia (Chen et al., 2014; So et al., 2017), obesity (Catapano et al., 2016), and metabolic syndrome (Haroon et al., 2014), which interact with AS and affect the occurrence of CVD complications in AS patients. Our MR analysis did not provide evidence for a causal effect of AS on AF, MI, Coronary atherosclerosis, Ischemic stroke, and VHD, which is consistent with previous MR study results (Chen et al., 2022; Mei et al., 2022).

Considering that heart failure typically arises as a consequence of various cardiovascular conditions, including atrial fibrillation, coronary artery disease, ischemic stroke, myocardial infarction, or valvular heart disease, the outcomes may be expounded upon through a multidimensional lens encompassing pathophysiological, genetic, pharmacotherapeutic, and methodological perspectives. For instance, AS may induce myocardial electrical and structural remodeling via processes such as inflammation, immune response, and myocardial fibrosis (Roth et al., 2020; Tsao et al., 2022), thereby elevating the risk of HF. Similar pathophysiological mechanisms are commonly observed in conditions like myocardial infarction (Roger et al., 2020), stroke (Toussirot, 2021; Ding et al., 2022; Kwon et al., 2022), arrhythmias (Tsai et al., 2015; Setyawan et al., 2021), and valvular heart disease (Burgess and Thompson, 2015; Zhao et al., 2020). Furthermore, it is well recognized that genetic factors play a crucial role in the development and progression of AS (Tsao et al., 2022). The presence of these specific genetic variants in AS patients may predispose them to an increased susceptibility to HF(Malik et al., 2018; Skrivankova et al., 2021a; Hartiala et al., 2021). Moreover, pharmacological treatment for AS may also influence the occurrence of HF. Guidelines suggest that reducing the inflammatory burden of AS through medication may have a favorable impact on patients’ CVD risk (Roth et al., 2020). However, a meta-analysis indicated that non-selective NSAIDs and selective cyclooxygenase-2 inhibitors may have adverse effects on cardiovascular outcomes in patients with inflammatory joint diseases. Finally, research findings may be constrained by the limited statistical power of Mendelian randomization studies, as well as the heterogeneity and confounding factors present in clinical research on disease subtypes. Therefore, larger-scale studies are needed to clarify the role of AS in specific disease subtypes and to determine whether biases in previous observational studies are due to confounding factors.

Prior clinical research on AS and VHD has been somewhat limited, primarily focusing on aortic valve regurgitation, with most studies supporting a correlation between AS and VHD. Prospective studies have shown a significantly increased hazard ratio for aortic valve regurgitation in AS compared to general population cohorts (Azevedo and Pecoits-Filho, 2010). In longitudinal retrospective cohort studies, AS subjects had a 1.63-fold increased risk of developing VHD compared to non-AS subjects, with statistically significant differences (Kim and Choi, 2021). Subgroup analyses for types of VHD revealed significantly elevated risks of mitral valve disease, aortic valve disease, and tricuspid valve disease in AS subjects compared to non-AS subjects (Kim and Choi, 2021). Moreover, valvular heart involvement appears to be more common in patients with longer disease duration (Haroon et al., 2015). Studies have found that among AS patients with a 15-year disease duration, 3.5% developed aortic valve insufficiency, while among those with a 30-year disease duration, 10% developed aortic valve insufficiency (Divecha et al., 2005). Additionally, retrospective cross-sectional studies indicate a significant increase in the risk of VHD with aging among AS patients after adjusting for gender and ethnicity (Papagoras et al., 2013). Consistent with previous MR studies (Fauci, 2015; Bhattad et al., 2022), this study did not suggest a significant causal relationship between AS and VHD, possibly due to limitations in sample size and testing statistical power of MR studies. Additionally, related research has found that the relationship between serum lipid levels and CVD risk in inflammatory joint diseases is nonlinear and may be contradictory (Zhang et al., 2018; Jiménez-Balderas et al., 2001; Toms et al., 2011). Traditional regression methods adjusting for confounding factors may not be applicable, necessitating the introduction of statistical methods such as polynomial regression and segmented regression in subsequent studies. Furthermore, studies have found that controlling disease activity may influence the onset of AS (Bengtsson et al., 2018; Siao et al., 2021), with most observational studies not incorporating this modifiable factor, posing challenges to research outcomes.

Many studies have shown that AS increases the morbidity and mortality of cardiovascular diseases (Graham and Smythe, 1958; Lui et al., 2011; Ward, 2018). However, the mechanism behind this association is still unclear. Research has shown elevated levels of pro-inflammatory cytokines such as IL-6 and CRP in AS (Zhong et al., 2023), suggesting that inflammation may play a role. Studies have shown that AS can lead to obliterative endarteritis in small vessels supplying the atrioventricular node and the aortic root, leading to a prolongation of the PQ interval and an increased risk of atrial fibrillation, first-degree atrioventricular block, and aortic valve insufficiency (Haroon et al., 2015). IL-6 induces the acute phase response, leading to increased levels of CRP and fibrinogen and monocyte activation, the activated monocytes deposit fibrinogen in the vessel wall, leading to atherosclerosis (Cai et al., 2024). Moreover, when the endothelium is damaged, foam cells and smooth muscle cells release IL-6 and other inflammatory cytokines, causing more vascular damage and exacerbating this process, ultimately leading to the formation and progression of atherosclerosis (Myasoedova et al., 2011). In addition, during the course of AS, IL-6 interacts with the hypothalamic-pituitary-adrenal (HPA) axis, leading to traditional cardiovascular risk factors including decreased insulin sensitivity, increased BMI, and the occurrence of hypertension (Boyer et al., 2012), exacerbating atherosclerosis.

It is worth noting that in the investigation of the association between AS and conduction abnormalities, a meta-analysis incorporating eight studies revealed a significantly heightened risk of atrioventricular conduction block among AS subjects compared to controls (Kerekes et al., 2009). Similarly, observational and retrospective cohort studies have reported such findings (Forsblad-d'Elia et al., 2013; Raterman et al., 2013; Morovatdar et al., 2021; Wu et al., 2016). However, another meta-analysis did not indicate a significant correlation between AS and cardiac conduction abnormalities (Hung et al., 2016). Regarding specific types of conduction abnormalities, prospective studies have shown a significantly elevated hazard ratio for II-III degree AV block and pacemaker implantation in AS compared to general population cohorts (Azevedo and Pecoits-Filho, 2010). Furthermore, research suggests that AS patients with arrhythmias exhibit higher disease activity indices compared to those without arrhythmias, with statistically significant differences, indicating a potential association between arrhythmias and AS disease activity (Dik et al., 2010). Mechanistically, arrhythmias often reflect the presence of cardiac conduction reentry resulting from inflammatory processes, fibromuscular proliferation and fibrosis, as well as increased automaticity and triggered activity (So et al., 2017). Additionally, the human leukocyte antigen B27 is recognized to potentially contribute to arrhythmogenesis by increasing platelet adhesion to vascular walls (Dalecky et al., 2024). Moreover, drug-induced arrhythmias, such as those caused by non-steroidal anti-inflammatory drugs (NSAIDs) and chloroquine, are commonly observed in AS (Moyssakis et al., 2009; Aksoy et al., 2016; Gawałko et al., 2020). NSAIDs, in particular, may affect cardiac electrophysiological properties by influencing various cardiac ion channels (Stas et al., 2008). As for myocardial disease, only limited literature exists on this topic (Haroon et al., 2015; Bakhriansyah et al., 2019), indicating the need for further investigation.

However, there are some limitations to this pilot study: At first, this study included individuals primarily of European descent, so extrapolating the study results to other populations is limited. Additionally, the study uses summary-level data, providing only binary variables, and the relationship between AS severity and CVD severity is still unclear. Furthermore, the odds ratio values obtained in this study are relatively lower compared to other studies, and further confirmation is needed in subsequent research. Therefore, we need to treat the results of MR with caution.

## Conclusion

In summary, this study suggests an increased risk of HF in patients with AS. The findings provide new genetic evidence for the causal relationship between AS and CVDs risk. Clinically, it is important to assess the cardiac function of AS patients and facilitate early prevention of cardiac dysfunction. Additionally, further exploration of the mechanisms underlying the development of HF in AS patients can aid in early diagnosis and treatment of cardiovascular complications.

## Data Availability

The original contributions presented in the study are included in the article/Supplementary Material, further inquiries can be directed to the corresponding author.

## Supplementary material

SUPPLEMENTARY FIGURE F1 Inverse Variance Weighted outcome of Mendelian randomization for the association between ankylosing spondylitis and the risks of cardiovascular disease. IVW: Inverse Variance Weighted method; MR, Mendelian randomization; SNPs, single nucleotide polymorphisms; OR, odds ratio; CI, confidence interval.

SUPPLEMENTARY FIGURE F2 Mendelian randomization results for heart failure on ankylosing spondylitis risk.

SUPPLEMENTARY FIGURE F3 The scatter plot of Mendelian randomization methods for ankylosing spondylitis on cardiovascular disease. **(A)**, atrial fibrillation; **(B)**, coronary atherosclerosis; **(C)**, heart failure; **(D)**, ischemic stroke; **(E)**, myocardial infarction; **(F)**, valvular heart disease. Regression lines represent the causal effect of the ankylosing spondylitis on cardiovascular disease risk using IVW, MR-Egger, and Weighted median to estimate. MR: Mendelian randomization; SNPs: single nucleotide polymorphisms.

SUPPLEMENTARY FIGURE F4 Leave-one-out analysis for ankylosing spondylitis on cardiovascular disease. **(A)**, atrial fibrillation; **(B)**, coronary atherosclerosis; **(C)**, heart failure; **(D)**, ischemic stroke; **(E)**, myocardial infarction; **(F)**, valvular heart disease. MR: Mendelian randomization; SNPs: single nucleotide polymorphisms.

SUPPLEMENTARY FIGURE F5 Funnel plot from genetically predicted ankylosing spondylitis on cardiovascular disease. **(A)**, atrial fibrillation; **(B)**, coronary atherosclerosis; **(C)**, heart failure; **(D)**, ischemic stroke; **(E)**, myocardial infarction; **(F)**, valvular heart disease.
