# Supplementary material for: Association of ankylosing spondylitis with cardiovascular disease: a bidirectional two-sample mendelian randomization study
Source: Front Genet. 2024 Jun 26;15:1260247. doi: 10.3389/fgene.2024.1260247 (PMC11233527; doi:10.3389/fgene.2024.1260247)
Supplement: Supplementary file 1 [file Table1.DOCX]

**Supplementary Table S2**

Characteristics of the 13 candidate SNPs predictive of ankylosing spondylitis.

| **SNP**^†1^ | **EAF**^†2^ | **beta** | **SE**^†3^ | **N** | **R^2^** | **F**^†4^ |
| --- | --- | --- | --- | --- | --- | --- |
| rs13033284 | 0.6277 | -0.2214 | 0.0386 | 166144 | 1.98E-04 | 32.8984 |
| rs9264277 | 0.7297 | 0.5198 | 0.0448 | 166144 | 8.10E-04 | 134.6205 |
| rs9265893 | 0.1772 | 1.9426 | 0.0588 | 166144 | 6.53E-03 | 1091.4576 |
| rs34982906 | 0.0528 | 0.8211 | 0.0918 | 166144 | 4.81E-04 | 80.0021 |
| rs16894011 | 0.0727 | 2.1075 | 0.0886 | 166144 | 3.39E-03 | 565.7994 |
| rs9391773 | 0.1285 | 2.6349 | 0.0702 | 166144 | 8.41E-03 | 1408.7983 |
| rs62394289 | 0.1376 | 0.3738 | 0.0557 | 166144 | 2.71E-04 | 45.0363 |
| rs79693223 | 0.0450 | 1.2953 | 0.1062 | 166144 | 8.95E-04 | 148.7601 |
| rs9378220 | 0.2269 | -0.6933 | 0.0558 | 166144 | 9.28E-04 | 154.3721 |
| rs112733823 | 0.2165 | 0.3600 | 0.0468 | 166144 | 3.56E-04 | 59.1709 |
| rs76644067 | 0.0475 | 0.7328 | 0.0946 | 166144 | 3.61E-04 | 60.0044 |
| rs181316459 | 0.0472 | 0.9847 | 0.1004 | 166144 | 5.79E-04 | 96.1912 |
| rs10807943 | 0.9365 | -0.5628 | 0.0812 | 166144 | 2.89E-04 | 48.0387 |

^†^1 SNP: rs ID; ^†^2 EAF: effect allele frequency; ^†^3 SE: standard error; ^†^4 F: F-statistics.
