# Supplementary figures and images for: Association of ankylosing spondylitis with cardiovascular disease: a bidirectional two-sample mendelian randomization study
Source: Front Genet. 2024 Jun 26;15:1260247. doi: 10.3389/fgene.2024.1260247 (PMC11233527; doi:10.3389/fgene.2024.1260247)

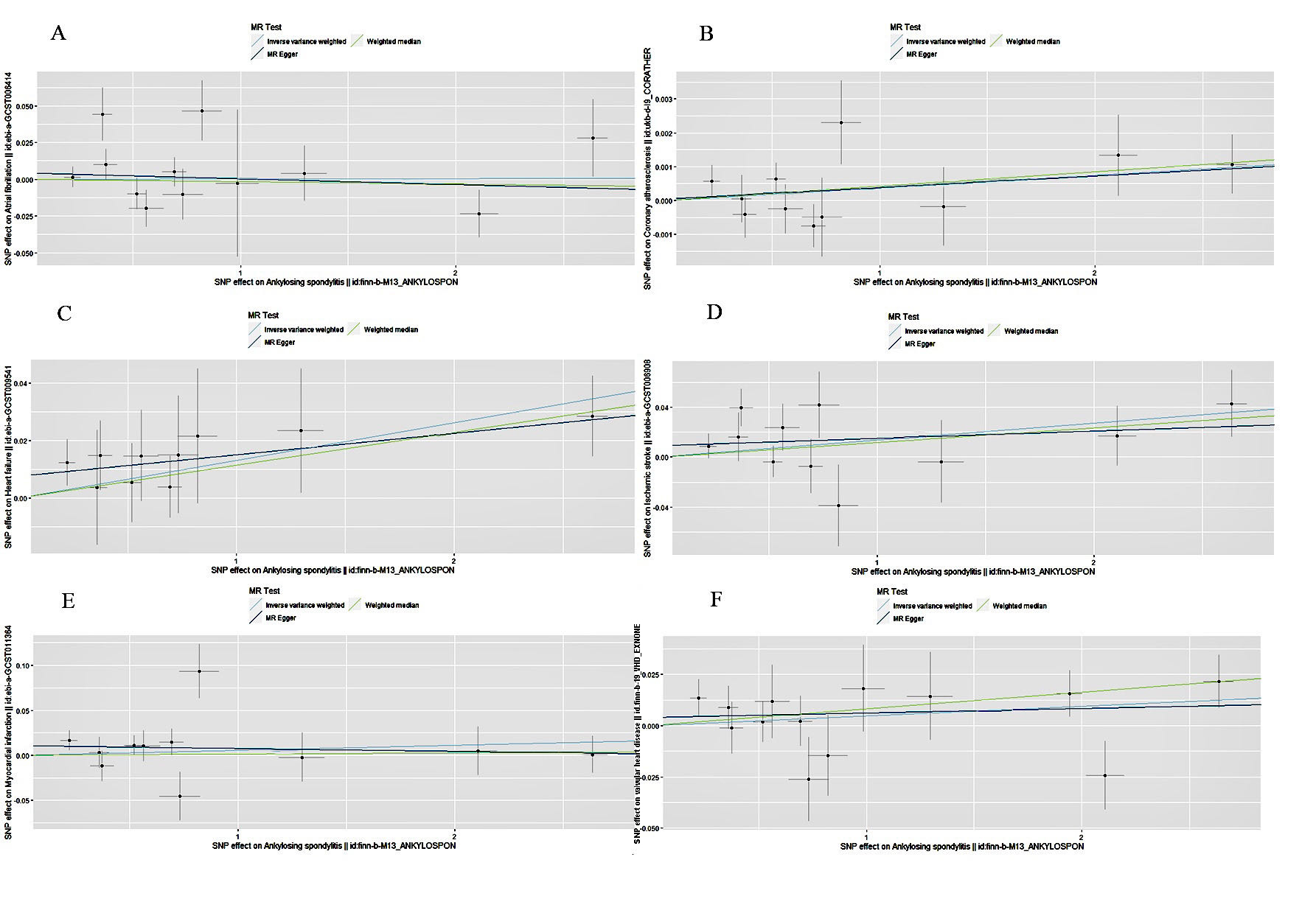

Supplement: Supplementary file 2 [file Image3.TIF]

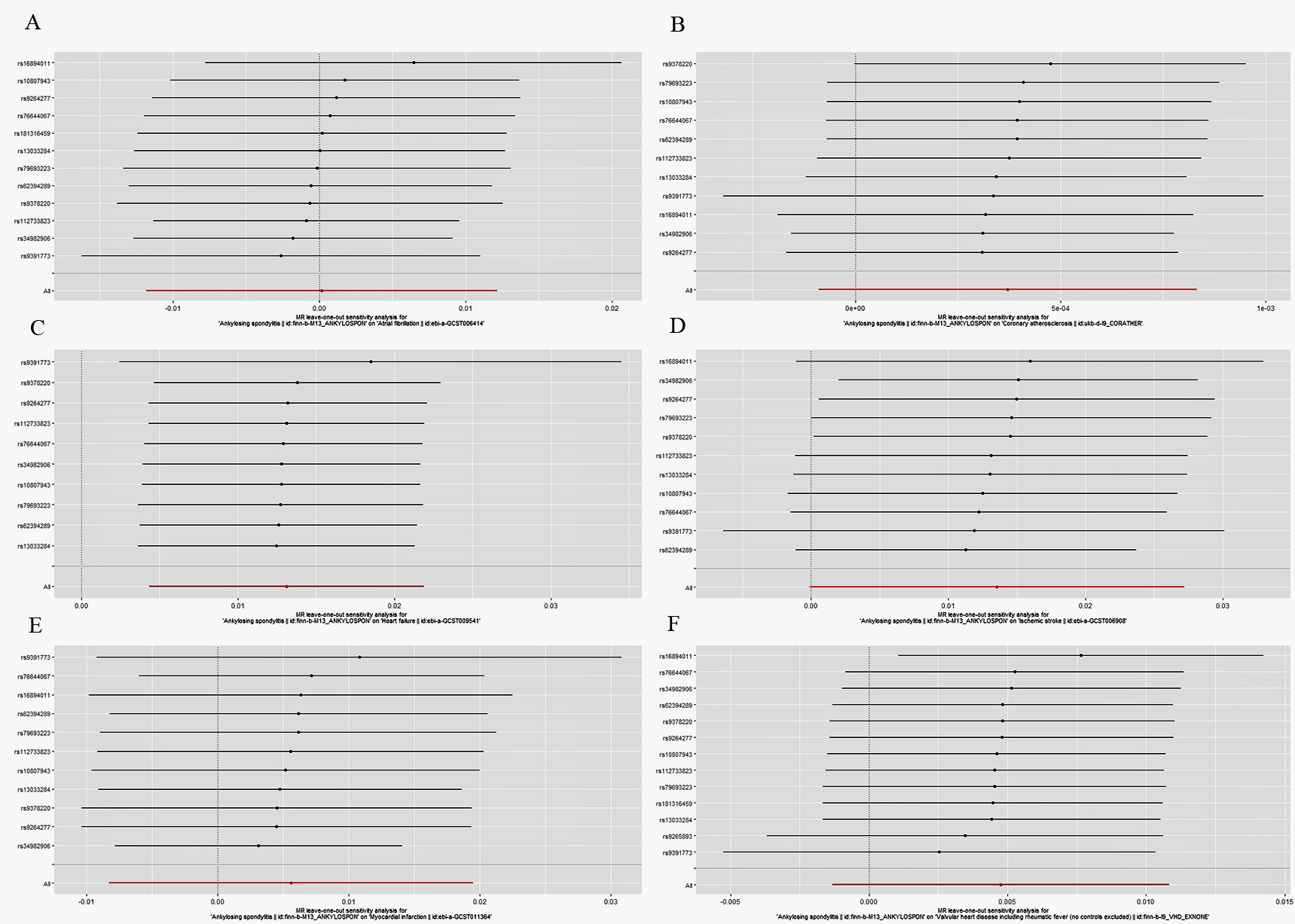

Supplement: Supplementary file 3 [file Image4.TIF]

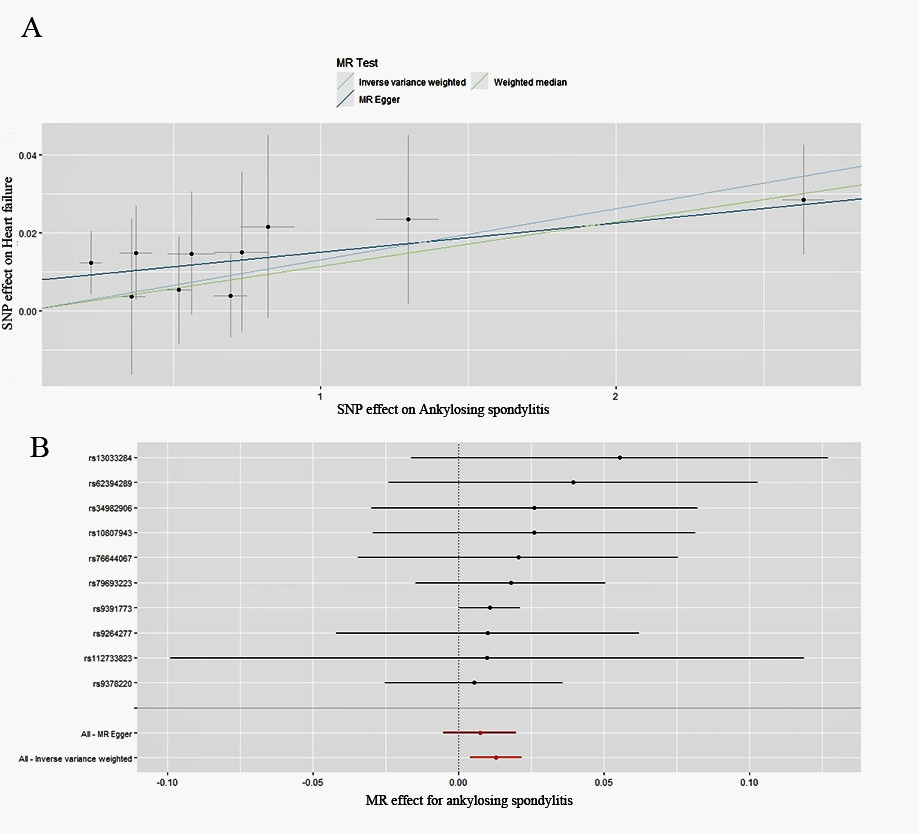

Supplement: Supplementary file 4 [file Image2.TIF]

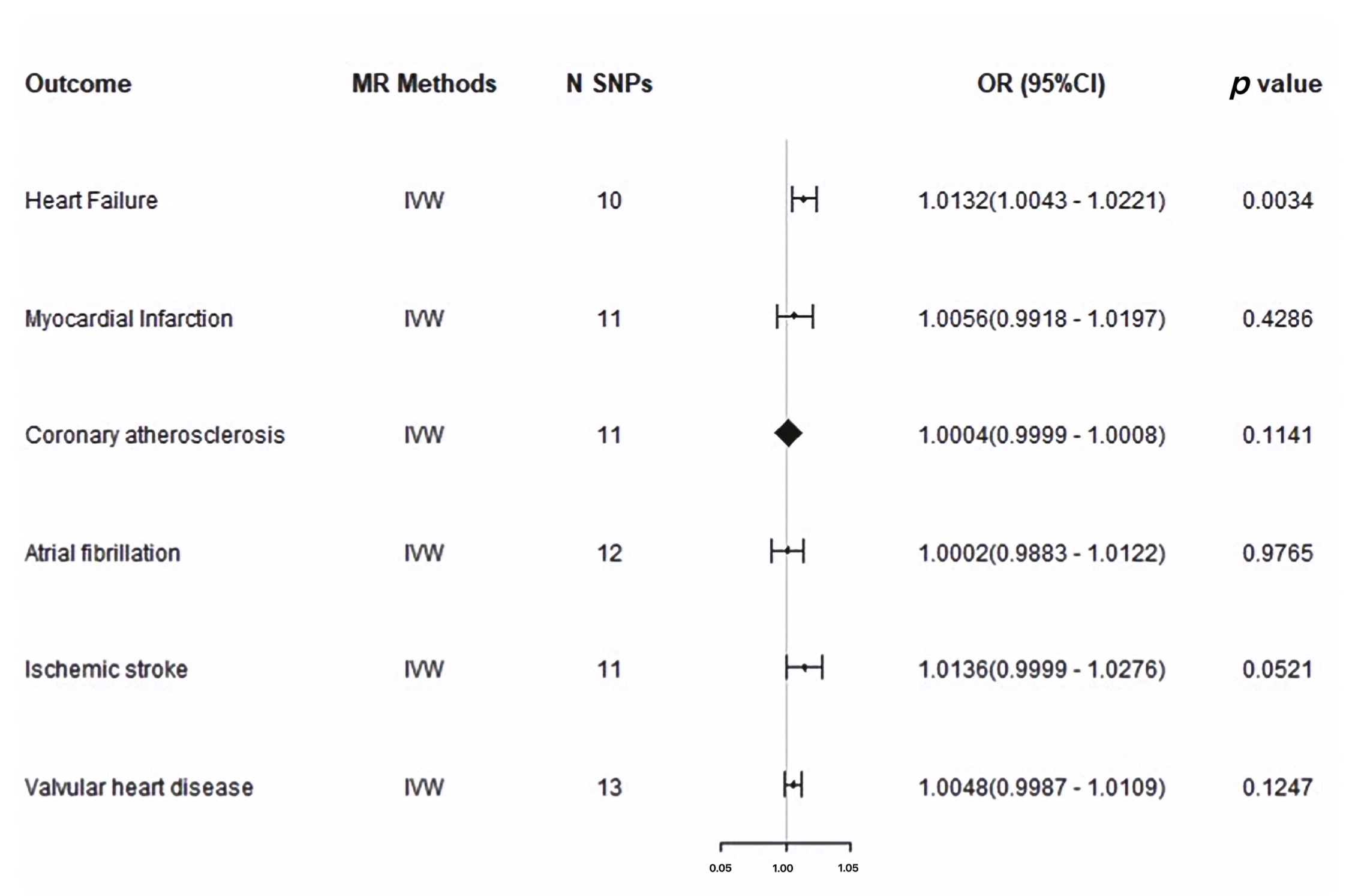

Supplement: Supplementary file 5 [file Image1.TIF]

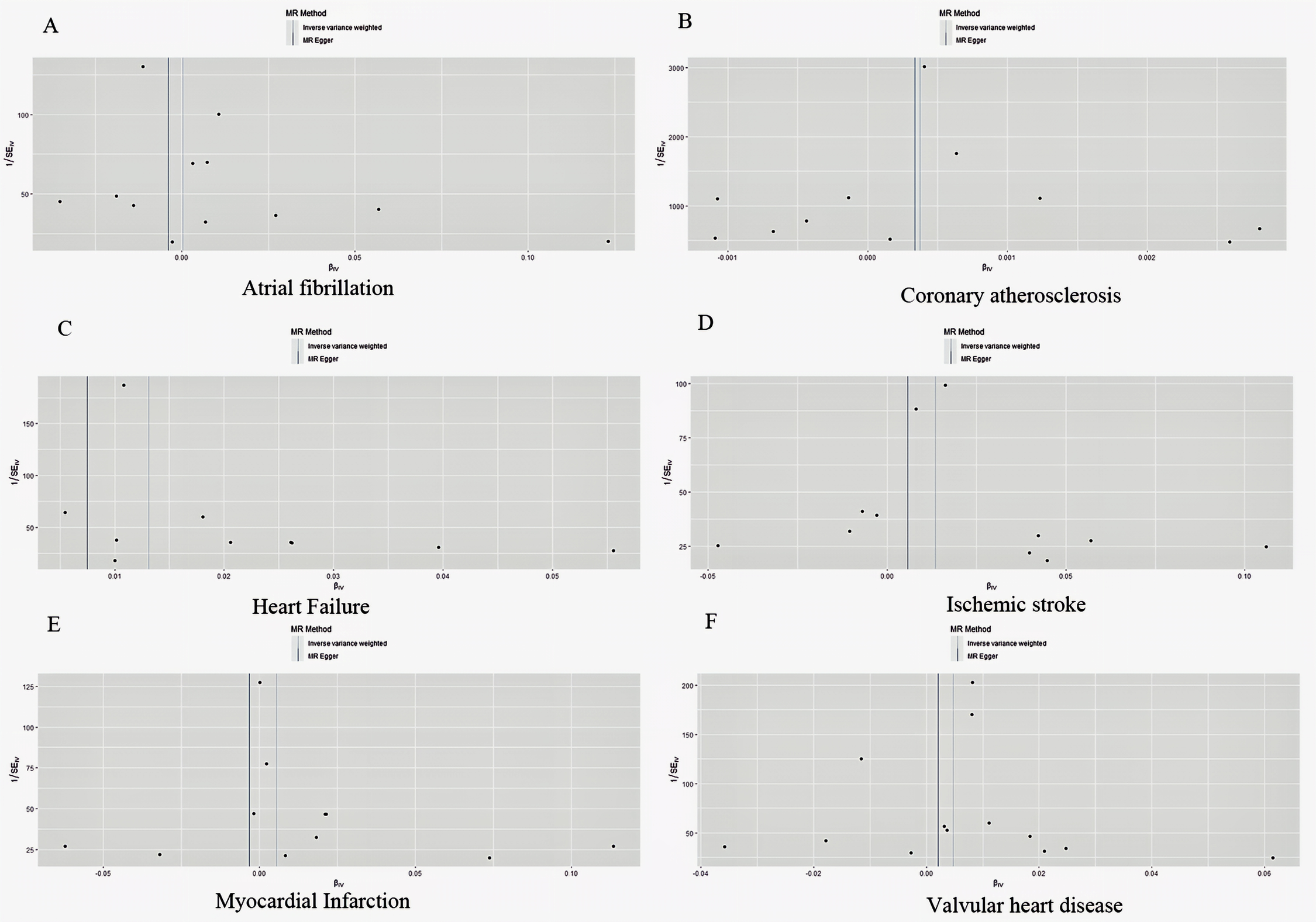

Supplement: Supplementary file 9 [file Image5.TIF]
