# Supplementary material for: Association of ankylosing spondylitis with cardiovascular disease: a bidirectional two-sample mendelian randomization study
Source: Front Genet. 2024 Jun 26;15:1260247. doi: 10.3389/fgene.2024.1260247 (PMC11233527; doi:10.3389/fgene.2024.1260247)
Supplement: Supplementary file 6 [file Table2.DOCX]

**Supplementary Table S2**

Mendelian randomization results for ankylosing spondylitis on cardiovascular disease risk.

| **Outcome** | **MR**^†2^ **Methods** | **N SNPs**^†3^ | **OR**^†4^ **(95%CI**^†5^**)** | **Se** | **P value** |
| --- | --- | --- | --- | --- | --- |
| Heart Failure | MR Egger | 10 | 1.0075(0.9949 - 1.0202) | 0.0064 | 0.2773 |
|  | IVW^†1^ | 10 | 1.0132(1.0043 - 1.0221) | 0.0045 | 0.0034 |
|  | Weighted median | 10 | 1.0115(1.0013 - 1.0217) | 0.0052 | 0.0274 |
| Myocardial Infarction | MR Egger | 11 | 0.9969(0.9769 - 1.0173) | 0.0104 | 0.7694 |
|  | IVW | 11 | 1.0056(0.9918 - 1.0197) | 0.0071 | 0.4286 |
|  | Weighted median | 11 | 1.0012(0.9879 - 1.0146) | 0.0068 | 0.8623 |
| Coronary atherosclerosis | MR Egger | 11 | 1.0003(0.9997 - 1.0010) | 0.0004 | 0.3605 |
|  | IVW | 11 | 1.0004(0.9999 - 1.0008) | 0.0002 | 0.1141 |
|  | Weighted median | 11 | 1.0004(0.9999 - 1.0010) | 0.0003 | 0.1383 |
| Atrial fibrillation | MR Egger | 12 | 0.9961(0.9773 - 1.0153) | 0.0097 | 0.6982 |
|  | IVW | 12 | 1.0002(0.9883 - 1.0122) | 0.0061 | 0.9765 |
|  | Weighted median | 12 | 0.9984(0.9860 - 1.0109) | 0.0064 | 0.7959 |
| Ischemic stroke | MR Egger | 11 | 1.0058(0.9862 - 1.0258) | 0.0100 | 0.5764 |
|  | IVW | 11 | 1.0136(0.9999 - 1.0276) | 0.0070 | 0.0521 |
|  | Weighted median | 11 | 1.0118(0.9965 - 1.0274) | 0.0078 | 0.1325 |
| Valvular heart disease | MR Egger | 13 | 1.0021(0.9928 - 1.0116) | 0.0048 | 0.6620 |
|  | IVW | 13 | 1.0048(0.9987 - 1.0109) | 0.0031 | 0.1247 |
|  | Weighted median | 13 | 1.0081(1.0003 - 1.0160) | 0.0040 | 0.0416 |

^†^1 IVW: Inverse Variance Weighted method. ^†^2 MR, Mendelian randomization; ^†^3 SNPs, single nucleotide polymorphisms. ^†^4 OR, odds ratio; ^†^5 CI, confidence interval.
