# Supplementary material for: Association of ankylosing spondylitis with cardiovascular disease: a bidirectional two-sample mendelian randomization study
Source: Front Genet. 2024 Jun 26;15:1260247. doi: 10.3389/fgene.2024.1260247 (PMC11233527; doi:10.3389/fgene.2024.1260247)
Supplement: Supplementary file 7 [file Table3.DOCX]

**Supplementary Table S3**

Mendelian randomization results for cardiovascular disease on ankylosing spondylitis risk.

| **Exposure** | **MR**^†2^ **Methods** | **N SNPs**^†3^ | **OR**^†4^ **(95%CI)** ^†5^ | **Se** | **P value** |
| --- | --- | --- | --- | --- | --- |
| Heart Failure | MR Egger | 9 | 0.8684(0.6636 - 1.4832) | 0.3942 | 0.8684 |
|  | IVW^†1^ | 9 | 0.8361(0.4816 - 1.4517) | 0.2815 | 0.5249 |
|  | Weighted median | 9 | 0.8619(0.4428 - 1.6775) | 0.3398 | 0.6618 |
| Myocardial Infarction | MR Egger | 30 | 0.9714(0.6017 - 1.5681) | 0.2443 | 0.9063 |
|  | IVW | 30 | 1.0936(0.8894 - 1.3448) | 0.1055 | 0.3960 |
|  | Weighted median | 30 | 1.0405(0.8033 - 1.3477) | 0.1320 | 0.7636 |
| Coronary atherosclerosis | MR Egger | 29 | 0.0262(7.737×10^-7^ -889.6537) | 5.3222 | 0.4998 |
|  | IVW | 29 | 5.4668(0.0650 - 459.6306) | 2.2611 | 0.4525 |
|  | Weighted median | 29 | 0.2518 (0.0007 - 92.7564) | 3.0149 | 0.6473 |
| Atrial fibrillation | MR Egger | 109 | 0.9042(0.7337 - 1.1142) | 0.1066 | 0.3467 |
|  | IVW | 109 | 0.9994(0.8962 - 1.1145) | 0.0556 | 0.9917 |
|  | Weighted median | 109 | 0.9541(0.7844 - 1.1605) | 0.0999 | 0.6381 |
| Ischemic stroke | MR Egger | 8 | 0.7315(0.0461 - 11.6083) | 1.4104 | 0.8319 |
|  | IVW | 8 | 0.8816(0.6030 - 1.2888) | 0.1938 | 0.5153 |
|  | Weighted median | 8 | 0.9264(0.5672 - 1.5129) | 0.2503 | 0.7599 |
| Valvular heart disease | MR Egger | 3 | 1.3516(0.1366 - 13.3691) | 1.1692 | 0.8395 |
|  | IVW | 3 | 0.8449(0.4052 - 1.7619) | 0.3749 | 0.6532 |
|  | Weighted median | 3 | 0.8126(0.3667 - 1.8008) | 0.4060 | 0.6092 |

^†^1 IVW: Inverse Variance Weighted method. ^†^2 MR, Mendelian randomization; ^†^3 SNPs, single nucleotide polymorphisms. ^†^4 OR, odds ratio; ^†^5 CI, confidence interval.
