# Supplementary material for: Association of ankylosing spondylitis with cardiovascular disease: a bidirectional two-sample mendelian randomization study
Source: Front Genet. 2024 Jun 26;15:1260247. doi: 10.3389/fgene.2024.1260247 (PMC11233527; doi:10.3389/fgene.2024.1260247)
Supplement: Supplementary file 8 [file Table4.DOCX]

**Supplementary Table S4**

Sensitivity analysis of the reverse MR analysis results of exposures and outcomes.

| **Exposure** | **Pleiotropy test**  **MR-egger** |  |  | **Heterogeneity test**  **MR-egger** |  | **IVW**^†3^ |  | **MR-PRESSO global test** | |
| --- | --- | --- | --- | --- | --- | --- | --- | --- | --- |
|  | **Intercept** | **SE**^†1^ | **P-value**^†2^ | **Cochran’s Q** | ***P*-value** | **Cochran’s Q** | ***P*-value** | **RSS obs**^†4^ | **P-value** |
| HF^†5^ | -0.039 | 0.056 | 0.506 | 9.565 | 0.215 | 10.236 | 0.249 | 13.543 | 0.395 |
| MI^†6^ | 0.014 | 0.025 | 0.594 | 45.819 | 0.018 | 46.295 | 0.022 | 49.189 | 0.016 |
| Coronary atherosclerosis | 0.028 | 0.025 | 0.278 | 35.859 | 0.118 | 37.488 | 0.108 | 45.059 | 0.042 |
| AF^†7^ | 0.009 | 0.008 | 0.273 | 114.077 | 0.302 | 115.370 | 0.296 | 117.617 | 0.332 |
| IS^†8^ | 0.014 | 0.107 | 0.898 | 6.010 | 0.422 | 6.028 | 0.537 | 7.677 | 0.527 |
| VHD^†9^ | -0.033 | 0.077 | 0.745 | 4.08×10^-4^ | 0.984 | 0.180 | 0.914 | 49.189 | 0.016 |

^†^1 SE: standard error; ^†^2 P-value < 0.05 was considered as with statistical differences in both heterogeneity and horizontal pleiotropy tests; ^†^3 IVW: Inverse Variance Weighting. ^†^4 RSS, residual sum of squares. ^†^5 HF: Heart Failure; ^†^6 MI: Myocardial Infarction; ^†^7 AF: Atrial fibrillation; ^†^8 IS: Ischemic stroke; ^†^9 VHD: Valvular heart disease
